# Association between overweight and anemia in Moroccan adolescents: a cross-sectional study

**DOI:** 10.11604/pamj.2022.41.156.20927

**Published:** 2022-02-22

**Authors:** Slimane Mehdad, Souad Benaich, Asmaa El Hamdouchi, Nezha Bouhaddou, Mehdi Azlaf, Imane El Menchawy, Hakim Belghiti, Hasnae Benkirane, Houria Lahmam, Amina Barkat, Khalid El Kari, Mohamed El Mzibri, Hassan Aguenaou

**Affiliations:** 1 Physiology and Physiopathology Team, Faculty of Sciences, Mohammed V University, Rabat, Morocco,; 2 Joint Research Unit in Nutrition and Food, RDC-Nutrition AFRA/IAEA, Ibn Tofail University-CNESTEN, Rabat-Kenitra, Morocco,; 3 Mohammed V Military Hospital of Instruction, Rabat, Morocco,; 4 Unit of Research on Nutrition and Health of Mother and Child, Faculty of Medicine and Pharmacy, Mohammed V University, Rabat, Morocco

**Keywords:** Adolescents, anemia, body-fat, hemoglobin, obesity, overweight

## Abstract

**Introduction:**

obesity and anemia remain global public health problems, having major negative effects on human health. We aimed to estimate the prevalence of anemia and investigate its association with overweight/obesity and excess body fat among Moroccan adolescents.

**Methods:**

a total of 292 adolescents aged 11-17 years were recruited. Body mass index (BMI) and waist circumference (WC) were determined using standardized equipment. Body fat mass was measured by bioelectrical impedance analysis. Hemoglobin concentration was measured by the HemoCue method.

**Results:**

the overall prevalence of anemia was 13.7%. Anemia was more common among overweight/obese participants (15.2%) compared to non-overweight participants (12.8%). Overweight/obese boys were more likely to experience anemia than their non-overweight peers (odds ratio (OR): 1.49; 95% confidence interval (95%CI): 0.51-4.41). Similarly, anemia likelihood was higher among individuals having excess body fat than those who do not have excess body fat, particularly among girls (OR: 1.64; 95%CI: 0.69-3.87). Excess body fat was also significantly associated with lower hemoglobin concentration in both the total sample and girls (P=0.014, and P=0.041, respectively). Overall, increased BMI, WC, fat mass, and percent body fat were associated with reduced hemoglobin concentrations. There was a significant negative correlation between hemoglobin concentration and BMI among anemic girls (P=0.023).

**Conclusion:**

elevated BMI and body fat level were associated with lower hemoglobin concentrations and anemia. Further studies are needed to delineate the basis of such associations, and if these findings are confirmed, the guidelines for screening for anemia may need to be modified to include overweight/obesity as a risk factor.

## Introduction

Over the last decades, obesity has become one of the most common health problems in both developed and developing countries. Excess body fat accumulation may lead to serious long-term health consequences in all age groups [1]. Among children and adolescents, obesity is a recognized risk factor for many comorbidities including cardiovascular diseases, diabetes mellitus, dyslipidemia, musculoskeletal disorders, asthma, sleep apnea, psychological problems, and some cancers [2]. Several studies have also reported significant associations of obesity and overweight with micronutrient deficiencies such as iron, vitamin D, and vitamin B12 [3-5], as well as with anemia [6-9]. There is a growing body of evidence that adolescents are at greater risk of developing anemia. According to the World Health Organization (WHO) data, approximately 30% to 55% of adolescents worldwide are estimated to be anemic [10]. In this age group, girls are more likely to have anemia than boys [11-13] due to menstrual blood loss, faster body growth [14,15], and insufficient iron in their diets [16]. The impact of anemia on adolescents´ health is well known. It affects physical growth and mental development and subsequently decreases learning ability and school performance. It may also lead to other adverse effects such as decreased levels of energy and impaired immune function [15,17].

As in many low- and middle-income countries, rapid dietary and lifestyle changes in Morocco have led to a double burden of malnutrition with under- and over-nutrition in the same population [18,19]. Previous population-based surveys revealed that 32.6% of women aged 15-49 years, 31.5% of children under 5 years, and 18.0% of men aged 18-60 years were found to be anemic [20], while 32.9% and 17.9% of adults aged 20 years and older were overweight and obese, respectively [21]. Thus, obesity and anemia are major public health burdens in the country, and their coexistence may have a greater negative impact than either one alone. In Morocco, research on the prevalence of anemia and its associated factors among adolescents is still scarce, and the investigation focusing on this age group is not yet included in large epidemiologic studies and national surveys. Moreover, only a few studies explored the role of excess body fat as a risk factor for anemia. Some of these studies reported a significant association between obesity and anemia in adolescents [6,7], while others did not find any association [22,23]. Thus, more research is needed to identify adolescents with increased risk profiles and to provide relevant data to design appropriate interventions for preventing and controlling anemia. The current study aimed to estimate the prevalence of anemia in a sample of Moroccan adolescents and to investigate, for the first time to our knowledge, its association with weight status and body fat levels.

## Methods

### Study design and participants

This cross-sectional study was carried out in ten public secondary schools randomly selected from Rabat region, Morocco, during the academic year 2018-2019. The sample size (n) was determined using the formula developed by Cochran (1977) [24]: n = z^2^× p × (1 - p) / m^2^ where z = confidence level according to the normal distribution (for a 95% confidence interval, z = 1.96); p = estimated prevalence of anemia among Moroccan adolescents (based on the average prevalence of anemia reported in two previous studies conducted by Aboussaleh *et al*. [25] and Achouri *et al*. [26], the estimated p = 24%); and m = 5% (tolerated margin of error). Thus, 280 subjects were required to obtain statistically representative data. The total number of adolescents who were selected randomly and met the inclusion criteria was 292. Before data collection, principals and teachers of life and earth sciences in targeted schools were informed about the study and were invited to facilitate the recruitment of participants. Written informed consent of parents and verbal assent were obtained from each student involved in the study. A brief clinical examination was performed by a pediatrician. Students having disabilities or known chronic diseases were not included. The study protocol was approved by the research ethic board of Ibn Tofail University-Kenitra, Morocco.

### Data collection

***Anthropometric measures:*** weight and height were measured by trained staff using standardized equipment (Seca Model 770 digital electronic scale, and height bar / 2 m -dismantling). The weight status of each subject was determined using the World Health Organization (WHO) - Body mass index (Kg/m^2^)-for-age criteria [27].

***Blood sampling and hemoglobin analysis:*** in the morning after an overnight fast, capillary blood samples were obtained by the finger-prick method using disposable lancets. Hemoglobin (Hb) concentration was determined on-site by the HemoCue® hemoglobinometer [28]. The whole procedure was performed by trained operators. The presence or absence of anemia was detected based on the WHO criteria for anemia [17].

***Body fat measurement:*** body composition was determined by bioelectrical impedance analysis using BIA-4000-Bodystat Quadscan [29]. The body fat level was considered excessive when percent body fat is greater than McCarthy´s 85^th^ percentile of body fat reference data [30].

### Statistical analysis

The Kolmogorov-Smirnov test was used to check if variables follow a normal distribution or not. Data were presented as mean ± standard deviation (normal distribution), median and interquartile range (non-normal distribution), and percentages for categorical variables using descriptive statistics. Two-way ANOVA was used to examine the effect of gender, weight status, and the interaction between gender and weight status. General linear model and logistic regression analyses were used to investigate the association of overweight/obesity and excess body fat with hemoglobin concentration and anemia, respectively. To assess the relationship between obesity measures and hemoglobin level, we used the Spearman rank correlation method when the studied variables are normally or non-normally distributed, and linear regression analysis when the variables do not have the same kind of distribution. P-values < 0.05 were considered statistically significant. All statistical analyses were undertaken using the software statistical package for social sciences (SPSS, version 20.0).

## Results

Characteristics of the study population are presented in Table 1. A total of 292 adolescents (196 girls and 96 boys) aged 11-17 years were recruited. As we had a low proportion of underweight and obese adolescents among the study sample, the weight status is presented in our analysis as a binary variable (non-overweight and overweight/obese). In this sample, 61.6% were non-overweight and 38.4% were either overweight or obese.

**Table 1:** characteristics of the study population

Variables	Gender	Total	Non-Overweight	Overweight/Obese	P Values		
		Girls: n=196 Boys: n=96	Girls: n=130 Boys: n=50	Girls: n=66 Boys: n=46	Effect of gender	Effect of weight status	Effect of interaction between gender and weight status
Age (years)	Girls	14.13 (14.23 - 14.86)	13.95 (13.12 - 14.79)	14.36 (13.56 - 15.20)	0.319	0.865	0.09
	Boys	14.11 ± 0.99	14.24 (13.60 - 15.05)	13.91 ± 0.93			
Weight (kg)	Girls	53.65 ± 14.37	46.00 (40.75 - 51.25)	68.00 (61.88 - 78.00)	0.956	< 0.001	0.009
	Boys	56.50 (47.05 - 67.50)	48.50 (41.00 - 57.50)	64.00 (56.00 - 71.25)			
Height (m)	Girls	1.58 ± 0.07	1.58 (1.51 - 1.62)	1.60 (1.56 - 1.64)	0.031	0.594	0.030
	Boys	1.61 (1.52 - 1.70)	1.61 (1.53 - 1.71)	1.61 (1.50 - 1.68)			
BMI (kg/m2)	Girls	21.35 ± 4.87	18.54 (16.78 - 20.26)	26.43 (24.02 - 29.26)	0.064	< 0.001	0.031
	Boys	22.15 (18.32 - 24.89)	18.43 (16.62 - 20.46)	25.11 (23.36 - 26.62)			
WC (cm)	Girls	74.95 ± 11.93	68.86 ± 7.69	86.50 (80.75 - 94.00)	0.006	< 0.001	0.589
	Boys	80.00 (71.00 - 88.00)	71.25 (66.00 - 79.00)	89.43 ± 10.63			
FM (kg)	Girls	15.43 ± 8.33	10.94 ± 4.48	24.26 ± 7.02	<0.001	< 0.001	0.022
	Boys	13.93 ± 7.95	9.15 ± 4.99	19.12 ± 7.30			
PBF (%)	Girls	26.61 ± 8.69	22.66 ± 7.01	34.51 (30.37 - 38.32)	<0.001	< 0.001	0.872
	Boys	22.87 ± 9.90	16.88 (14.07 - 21.91)	28.83 ± 8.88			
Hb (g/dL)	Girls	13.46 ± 1.41	13.54 ± 1.27	13.45 (12.48 - 14.43)	0.801	0.073	0.593
	Boys	13.60 (12.63 - 14.58)	13.69 ± 1.51	13.30 (12.18 - 14.55)			

(a) Data are mean ± standard deviation (SD) for variables normally distributed, medians (25 - 75% interquartiles) for variables non-normally distributed.

The overall prevalence of anemia was 13.7%. Anemia was more common among overweight/obese participants (15.2%) compared to non-overweight participants (12.8%) (Table 2). Overweight/obese boys were more likely to experience anemia than their non-overweight peers (odds ratio (OR): 1.49; 95% confidence interval (CI): 0.51-4.41). Similarly, individuals having excess body fat were more likely to have anemia compared to those who do not have excess body fat, particularly among girls (OR: 1.64; 95%CI: 0.69-3.87).

**Table 2:** odds ratio (OR) and 95% confidence interval (95%CI) of anemia according to weight status and body fat level

	Total				Boys				Girls			
	Anemia (%)	Crude OR a	95%CI a	P value	Anemia (%)	Crude OR a	95%CI a	P value	Anemia (%)	Crude ORa	95%CI a	P value
**Weight status**												
Non-overweight	12.8	1	13.36-13.79		14.0	1			12.3	1		
Overweight/obese	15.2	1.22	12.99-13.55	0.562	19.6	1.49	0.51-4.41	0.467	12.1	0.98	0.39-2.43	0.970
**Body fat level**												
Without excess	11.4	1	13.43-13.89		14.9	1			9.9	1		
With excess	16.4	1.53	12.98-13.48	0.215	18.4	1.29	0.44-3.79	0.649	15.3	1.64	0.69-3.87	0.258

**a**Crude odds ratio (OR) and 95% confidence interval (95%CI) using logistic regression.

Overweight/obesity and excess body fat were also associated with low hemoglobin concentration in the total sample, with a P-value of 0.092 and 0.014, respectively (Table 3). The hemoglobin level followed a similar trend among both genders separately. Girls having excess body fat showed significantly lower hemoglobin concentrations than their peers who do not have excess body fat (P = 0.041).

**Table 3:** mean and 95% confidence interval (95%CI) of hemoglobin concentration according to weight status and body fat level

	Total			Boys			Girls		
	Crude mean of Hb (g/dL) a	95%CI a	P value	Crude mean of Hb (g/dL) a	95%CI a	P value	Crude mean of Hb (g/dL)a	95%CI a	P value
**Weight status**									
Non-overweight	13.58	13.36-13.79	0.092	13.69	13.22-14.16	0.196	13.54	13.29-13.78	0.261
Overweight/obese	13.27	12.99-13.55		13.24	12.75-13.73		13.29	12.95-13.64	
**Body fat level**									
Without excess	13.66	13.43-13.89	0.014*	13.72	13.23-14.19	0.169	13.64	13.37-13.89	0.041*
With excess	13.23	12.98-13.48		13.24	12.77-13.72		13.22	12.92-13.52	

a Crude mean and 95%CI using general linear model. * Difference between categories significant at P < 0.05.

To check our hypothesis that overweight and excess body fat may predispose to anemia, we determined the correlation coefficient of hemoglobin concentration with each of body mass index (BMI), waist circumference (WC), fat mass (FM), and percent body fat (PBF). Overall, statistical analyses showed a negative correlation between these measures in both genders (Table 4). A significant correlation was observed between hemoglobin concentration and BMI among anemic girls (P = 0.023). The correlation of hemoglobin concentration with BMI and FM also showed a borderline statistical significance in non-anemic girls (P = 0.058, and P = 0.074, respectively).

**Table 4:** correlation coefficient (r) of hemoglobin concentration with BMI, WC, FM and PBF

	Hemoglobin concentration (g/dL)							
	BMI (Kg/m2)		WC (cm)		FM (Kg)		PBF (%)	
	r	P-value	r	P-value	r	P-value	r	P-value
**Boys**								
Total (n=96)	-0.101	0.329	-0.073	0.479	-0.003	0.979	-0.047	0.647
Non-Anemic (n=80)	-0.005	0.963	0.065	0.568	0.040	0.728	-0.103	0.363
Anemic (n=16)	-0.248	0.354	-0.011	0.969	-0.214	0.425	-0.223	0.406
**Girls**								
Total (n=196)	-0.114	0.110	-0.097	0.176	-0.107	0.136	-0.042	0.556
Non-Anemic (n=172)	-0.145	0.058	-0.109	0.154	-0.137	0.074	-0.061	0.429
Anemic (n=24)	-0.461	0.023*	-0.336	0.109	-0.232	0.276	-0.200	0.350

* Correlation significant at P < 0.05

## Discussion

Among our study population, anemia prevalence was 13.7%. It was slightly higher among overweight/obese individuals as compared to their non-overweight peers (15.2 % vs. 12.8%). According to the WHO criteria for public health significance of anemia based on its prevalence [17], anemia would be classified as a mild public health problem in our sample.

Despite serious health implications of anemia, there is little information about its prevalence among Moroccan youth. One of the rare studies on anemia, conducted in Kenitra (Northwest of Morocco), indicated that 32% of adolescents aged 12-15 years were anemic [25]. Another one, carried out a few years later in the same region, found that 16.2% of schoolchildren aged 6-15 years had anemia [26]. Although it is difficult to compare our findings with those reported previously and with the WHO global estimates of anemia prevalence in adolescents (30-55%) [10], the proportion of anemic individuals was relatively low in our study sample. This is probably a result of various public health interventions performed over the last years to tackle micronutrient deficiencies, including food fortification and nutrition education [31,32].

Few studies have examined the association between anemia and obesity, particularly among adolescents, in whom these abnormalities are more common, and their coexistence may have an increased negative impact than either one alone. Some authors have reported that obesity is associated with micronutrient deficiencies and anemia [3,6,7], whereas other investigators have not found such an association. For instance, Kordas et al. reported in a study on 3267 Colombian females aged 13-49 years that overweight or obese individuals showed a lower prevalence of anemia compared to their normal-weight counterparts [22]. Another large cross-sectional study also revealed that hemoglobin concentration, as an indicator of anemia, was not lower in overweight and obese persons as compared to their peers in the normal weight range [23].

In this study, increased BMI was significantly correlated with low hemoglobin concentration in anemic girls; and overweight/obese persons had a higher likelihood of anemia than non-overweight persons. Moreover, individuals having excess body fat showed lower hemoglobin concentration than those having normal body fat levels in both the total sample (P = 0.014) and girls (P = 0.041). Overall, our results are consistent with those reported previously by other authors who found a significant association between overweight/obesity and both hemoglobin concentration [6,33] and anemia [7,34]. In addition, some studies have shown that iron deficiency anemia is more encountered among overweight adolescents than their normal-weight peers [35-37]. For example, in a cross-sectional study investigating the iron status among 321 children and adolescents, the highest proportion of subjects with iron deficiency was observed among overweight children (38.8%), followed by children at risk for overweight (12.1%), and normal-weight children (4.4%), with a statistically significant difference [35]. Another cross-sectional study on 1688 school children showed that BMI-for-age z-score was found to be a significant negative predictor of body iron stores [37].

The association of low hemoglobin concentration and anemia with overweight/obesity and excess body fat may be due to combined effects of nutritional and functional parameters. As iron deficiency remains the leading cause of anemia [38], many predisposing factors may enhance its occurrence in overweight/obese adolescents, such as inadequate iron intake due to restricted diets, reduced iron absorption in the small intestine, and greater iron requirement caused by a larger blood volume [39]. Anemia in obese individuals may be also caused by increased inflammatory activity in adipose tissue that leads to a higher production of hepcidin, a key hormone involved in several iron-related disorders [34,40]. In contrast, weight reduction can reduce inflammation and subsequently improve iron absorption in obese adolescents [41]. Furthermore, even when iron intake is adequate, overweight/obese individuals are at greater risk of iron deficiency due to its reduced bioavailability in their bodies [42]. Thus, anemia in overweight/obese adolescents requires more attention not only for its relatively high prevalence but also for its potential health consequences.

Some elements of the study design might have limitations to investigate the association of overweight and excess body fat with low hemoglobin concentration and anemia among adolescents. Firstly, the relatively small sample size may reduce the power of our study. Secondly, anemia may result from a multitude of factors including dietary intake, sexual maturation, and intestinal parasitic infections that were not addressed in this study. Thirdly, although hemoglobin concentration is the most reliable indicator of anemia compared to other measures, the study did not use iron indicators that may be helpful in data interpretation. Despite these limitations, our findings highlight the double burden of malnutrition, among Moroccan adolescents, that requires urgent public health intervention. Our results also provide additional information to update current knowledge on the epidemiology of anemia and its association with overweight and obesity. Finally, such findings could enable further studies on anemia as a national health concern that critically needs to be addressed.

## Conclusion

In conclusion, the risk of low hemoglobin concentration and anemia increased as BMI and body fat level increased. Further studies with larger sample sizes are required to generate data for a more comprehensive understanding of the association between overweight/obesity and anemia. Accordingly, if these findings are confirmed, the guidelines for screening for anemia may need to be modified to include excess body weight as one of its risk factors.

### What is known about this topic

- Anemia and obesity are serious public health problems having major consequences on human health, particularly in low- and middle-income countries;
- Overweight and obesity are recognized as risk factors for many metabolic and cardiovascular diseases, but few studies demonstrated their role as risk factors for micronutrient deficiencies and anemia;
- Some cross-sectional studies have reported a significant association between overweight/obesity and anemia; however, there is no consensus on the causal relationship between these two abnormalities, and further research is needed in various populations and ethnic groups.

### What this study adds

- This study provides an additional estimate of anemia prevalence among a sample of Moroccan adolescents aged 11-17 years and brings out the necessity to consider adolescents as one of the major risk groups for anemia
- Despite limitations, the study provides information on the association between overweight/obesity and anemia in Moroccan adolescents and highlights the double burden of malnutrition that requires urgent public health intervention;
- This research adds, to the body of knowledge on overweight/obesity and anemia, new data on the correlation between hemoglobin concentration and obesity measures including accurate fat mass and percent body fat among Moroccan adolescents (as an ethnic group from North Africa and Arab World).
